# Analysis of neutrophil-to-lymphocyte and platelet-to-lymphocyte ratios as inflammatory biomarkers in chronic kidney disease: impact of parathyroidectomy

**DOI:** 10.1590/2175-8239-JBN-2023-0175en

**Published:** 2024-04-08

**Authors:** Andre Kakinoki Teng, Eduardo Jorge Duque, Shirley Ferraz Crispilho, Wagner Domingues, Vanda Jorgetti, Luciene M. dos Reis, Rosilene M. Elias, Rosa Maria Affonso Moysés

**Affiliations:** 1 Universidade de São Paulo, Faculdade de Medicina, São Paulo, SP, Brazil.; 2 Universidade Nove de Julho, São Paulo, SP, Brazil.

**Keywords:** Renal Insufficiency, Chronic, Chronic Kidney Disease-Mineral and Bone Disorder, Hyperparathyroidism, Secondary, Parathyroidectomy

## Abstract

**Introduction:**

Secondary hyperparathyroidism (SHPT) is one of the causes for inflammation in CKD. We assessed the impact of parathyroidectomy (PTX) on neutrophil-to-lymphocyte (N/L) and platelet-to-lymphocyte (P/L) ratios in SHPT patients.

**Methods:**

A total of 118 patients [hemodialysis (HD, n = 81), and transplant recipients (TX, n = 37)] undergoing PTX between 2015 and 2021 were analyzed.

**Results:**

There was a significant reduction in calcium and PTH levels in both groups, in addition to an increase in vitamin D. In the HD group, PTX did not alter N/L and P/L ratios. In the TX group, there was a reduction in N/L and P/L ratios followed by a significant increase in total lymphocyte count.

**Conclusion:**

N/L and P/L ratios are not reliable biomarkers of inflammation in SHPT patients undergoing PTX. Uremia, which induces a state of chronic inflammation in dialysis patients, and the use of immunosuppression in kidney transplant recipients are some of the confounding factors that prevent the use of this tool in clinical practice.

## Introduction

Chronic kidney disease is associated to a systemic inflammatory state of multifactorial origin. High concentrations of parathyroid hormone (PTH) and fibroblast growth factor 23 (FGF-23), as well as changes in calcium, phosphorus and vitamin D observed in CKD-mineral and bone disease (CKD-MBD), possibly contribute to this condition^1,2^.

Neutrophil-to-lymphocyte (N/L) and platelet-to-lymphocyte (P/L) ratios are low-cost, highly accessible laboratory indicators that have been studied for their value as inflammatory and prognostic biomarkers in different scenarios, including cancer, cardiovascular and infectious diseases, among others^3,4^. An increase in these ratios indicates a proportional increase in pro-inflammatory cells (neutrophils and platelets) in relation to immune response regulators (lymphocytes).

In the context of CKD, studies suggest an association between N/L and P/L ratios with inflammation, cardiovascular mortality and the need for renal replacement therapy^5,6^. Tonyali et al.^7^ observed an association between N/L ratio and glomerular filtration rate (N/L ratio 2.14 ± 0.73 in healthy controls *versus* 3.53 ± 2.3 in the CKD group, defined as GFR < 60 mL/min/1.73 m^2^; p = .000).

However, there are few studies on the usefulness of these biomarkers in the specific context of CKD-MBD. In patients with secondary hyperparathyroidism due to CKD (SHPT), parathyroidectomy (PTX) is a surgical treatment option for refractory cases. The aim of this study was to assess the role of PTX on N/L and P/L ratios in SHPT patients.

## Methods

This retrospective study analyzed data from medical records of SHPT patients who underwent PTX at our facility. Between January 2015 and December 2021, 172 patients underwent surgery. After excluding cases with no available laboratory data, 118 cases remained for analysis. Complete blood count, platelet count and serum total calcium, ionized calcium, phosphorus, PTH and vitamin D levels were recorded and analyzed. For transplant patients, serum creatinine and glomerular filtration rate (GFR) data were collected using the CKD-EPI formula^8^. For comparison, we used as initial blood count the one obtained immediately before the surgery date. The post-PTX exam was collected around 9 months after surgery.

The patients were divided into two groups, both of which were analyzed separately: kidney transplant patients (TX group) and hemodialysis patients (HD group). Nine patients who received a kidney transplant during the period between pre- and post-PTX examinations were excluded from the analysis.

Continuous data were presented as mean ± standard deviation or median and percentiles (25th; 75th). The comparison between HD and TX groups was performed using the unpaired t-test or Mann-Whitney test, according to parametric or non-parametric data distribution, respectively. For comparison before and after PTX, both groups used the paired t-test or Wilcoxon test for variables with normal or non-normal distribution, respectively. The analysis was conducted using GraphPad Prism 9.3.1^®^ software (GraphPad Software, Inc., CA, USA).

## Results

### Total Group

A total of 118 patients were analyzed, with 67 (56.7%) being female. Mean age was 44.7 ± 13 years. As described in Table 1, there was a decrease in PTH, total and ionized calcium, followed by an increase in 25-vitamin D concentration. There was no variation in serum phosphorus. In the leukogram analysis, there was an increase in leukocytes and lymphocytes. The N/L ratio did not change significantly, but we observed a decrease in P/L ratio.

**Table 1:** Total group laboratory data

Total group	Pre-PTX	Post-PTX	p
**Total calcium (mg/dL)**	10.0 (9.3; 10.8)	8.8 (8.2; 9.6)	<0.0001
**Ionized calcium (mg/dL)**	5.3 (4.9; 5.7)	4.6 (4.2; 5.1)	<0.0001
**Phosphorus (mg/dL)**	4.6 (2.9; 6.1)	4.0 (3.1; 5.5)	0.1454
**PTH (pg/mL)**	1455 (493; 2081)	95 (51; 217)	<0.0001
**Vitamin D (ng/mL)**	27.0 (18.8; 32.4)	32.2 (26.4; 44.0)	<0.0001
**Leukocytes (cells/mm** ^3^ **)**	5900 (5000; 7900)	6400 (5175; 8125)	0.0358
**Lymphocyte (cells/mm** ^3^ **)**	1235 (1010; 1783)	1460 (1045; 1910)	0.0033
**Neutrophils (cells/mm** ^3^ **)**	3920 (2945; 4963)	4185 (3150; 5465)	0.1028
**Platelets (10** ^3^ **cells/mm** ^3^ **)**	190.0 (149.8; 243.3)	195.5 (172.8; 236)	0.0978
**N/L ratio**	2.88 (1.97; 3.97)	2.85 (2.12; 3.80)	0.3052
**P/L ratio**	143 (105; 194.7)	132.9 (102.4; 180.5)	0.0233

### HD Group

**Figure 1:**
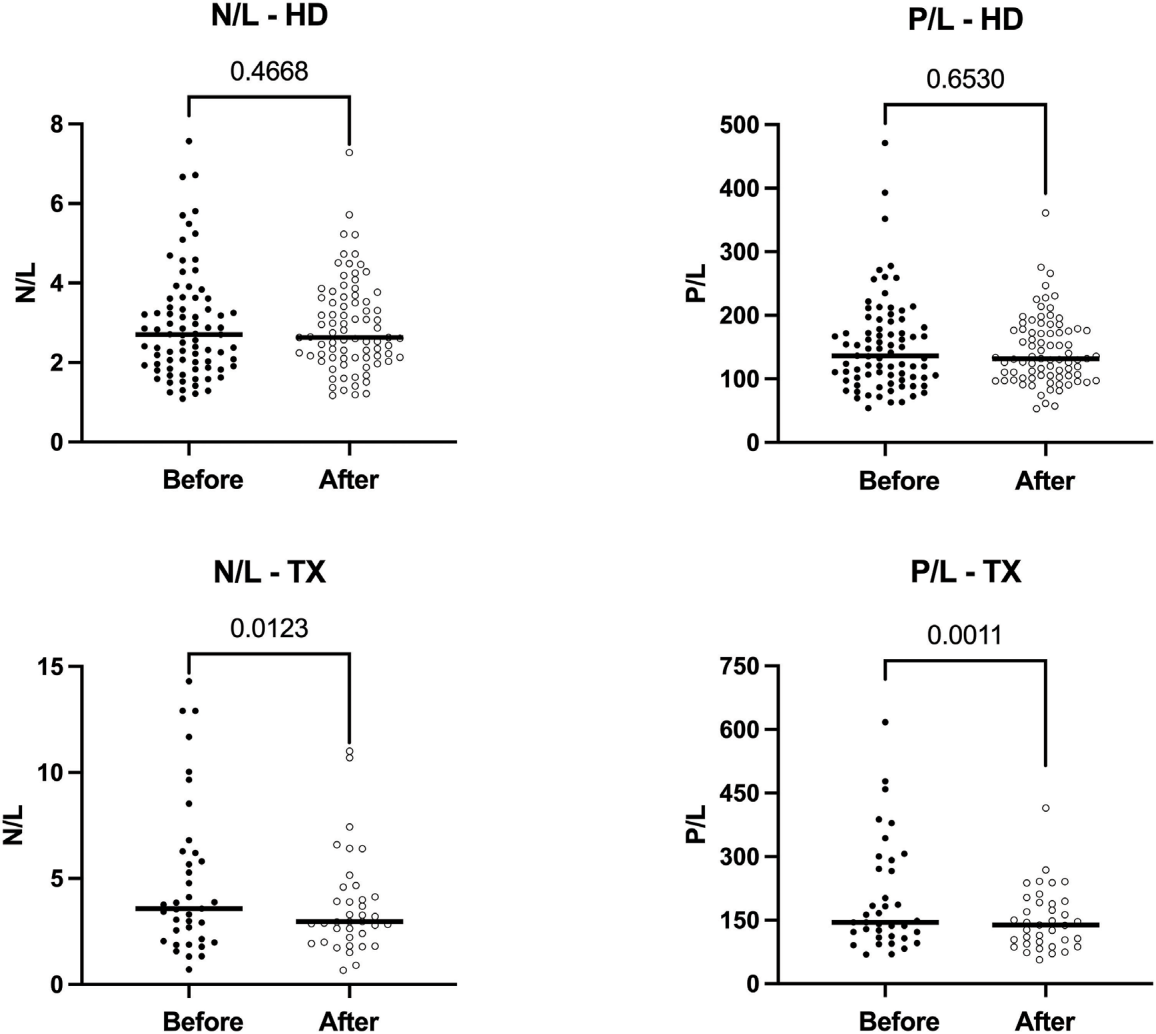
N/L and P/L ratios pre- and post-PTX - TX and HD groups.

In the HD group, 81 patients were analyzed, with 48 (59.2%) being female. Mean age was 42.5 ± 13.6 years. As shown in Table 2, there was a decrease in total calcium, ionized calcium, phosphorus, and PTH; there was also an increase in 25-vitamin D. The leukogram analysis showed a trend towards an increase in total leukocytes, but no significant difference in the total number of lymphocytes, neutrophils and platelets. No significant change was observed in the N/L and P/L ratios (Figure 1).

**Table 2:** HD and TX group laboratory data

	HD group			TX group		
	Pre-PTX	Post-PTX	p-Value	Pre-PTX	Post-PTX	p-Value
**Creatinine (mg/dL)**	–	–	–	1.43 (1.11; 1.91)	1.46 (1.13; 1.70)	0.1801
**GFR (mL/min/1.73 m** ^2^ **)**	–	–	–	49 (36; 59)	50 (32; 61)	0.6101
**Total calcium (mg/dL)**	9.6 (9.1; 10.4)	8.5 (7.6; 9.3)	<0.0001	10.5 (10.0; 9.4)	9.4 (9.0; 9.7)	<0.0001
**Ionized calcium (mg/dL)**	5,0 (4,7; 5,4)	4.4 (4.1; 4.7)	<0.0001	5.7 (5.5; 6.2)	5.0 (4.9; 5.4)	<0.0001
**Phosphorus (mg/dL)**	5,3 (4,5; 6,7)	4.8 (3.6; 5.9)	0.0002	2.5 (1.9; 2.9)	3.3 (2.7; 3.9)	<0.0001
**PTH (pg/mL)**	1695 (1417; 2509)	116 (43.5; 260)	<0.0001	294 (182; 536)	88 (57; 141)	<0.0001
**Vitamin D (ng/mL)**	29.0 (20.5; 35.05)	36.9 (28.4; 48.2)	<0.0001	22.6 (17.3; 28.7)	26.9 (25.1; 34.8)	0.0014
**Leukocytes (cells/mm** ^3^ **)**	5900 (5000; 8000)	6400 (4850; 8200)	0.069	5800 (4950; 7900)	6600 (5300; 8000)	0.2564
**Lymphocyte (cells/mm** ^3^ **)**	1370 (1060; 1950)	1500 (1150; 1855)	0.3137	1140 (795; 1645)	1280 (970; 1970)	<0.0001
**Neutrophils (cells/mm** ^3^ **)**	3900 (2940; 4765)	4170 (2895; 5545)	0.1116	3940 (3015; 5605)	4210 (3365; 5285)	0.6146
**Platelets (10** ^3^ **cells/mm** ^3^ **)**	198 (156; 248)	204 (175; 243.5)	0.202	174 (142; 201.5)	184 (158; 211.5)	0.2301
**N/L ratio**	2.7 (1.92; 3.61)	2.63 (2.12; 3.64)	0.4668	3.58 (2.09; 6.24)	2.97 (2.10; 4.36)	0.0123
**P/L ratio**	136.6 (101.8; 185.6)	131.7 (103.9; 177.4)	0.653	144.9 (110.7; 281.1)	138.6 (96.61; 193.2)	0.0011

### TX Group

In the TX group, 37 patients were analyzed, with 19 (51.3%) being female. Mean age was 49.7 ± 9.8 years. The mean interval between kidney transplantation and PTX was 21 months. As described in Table 2, a reduction in serum levels of PTH, total calcium and ionized calcium was observed. There was an increase in serum phosphorus and 25-vitamin D levels. There was no significant change in glomerular filtration rate and serum creatinine. A decrease was observed in the N/L and P/L ratios (Figure 1). This result is associated to an increase in lymphocyte count, with no significant change in the number of leukocytes, neutrophils and platelets.

## Discussion

SHPT is one of the contributing factors to the inflammatory state and cellular immune dysfunction observed in CKD patients. PTH is considered a uremic toxin, with known direct and indirect effects on inflammation, hematopoietic function, and adaptive immune response, both in lymphocytes and polymorphonuclear cells^9^. PTX, as a treatment option for SHPT refractory cases, has an effect on this inflammatory state, directly reducing the immunomodulatory effects of PTH.

In the HD group, PTX was associated with a significant reduction in PTH, calcium, and phosphorus, in addition to an increase in vitamin D, as expected, indicating its efficacy as a SHPT treatment. However, the analysis of results showed that there was no significant change in the N/L and P/L ratios in this group. This result may be related to the persistence of inflammatory state, multifactorial in origin, in CKD patients. Uremia, for instance, interferes through several mechanisms in the cellular immune response, both innate and adaptive^10^.

Accumulation of uremic toxins is linked to impaired function and proliferation of lymphocytes, which show increased apoptotic activity. Furthermore, exposure to uremia induces a process of immunosenescence, similar to what occurs with aging, with reduced lymphoproliferative activity in the thymus^11^. The neutrophil population, in turn, increases as GFR decreases. An increase in reactive oxygen species, myeloperoxidase, and priming activity is observed in HD patients, indicating a state of chronic inflammatory activation^12^. All these phenomena together are likely to contribute to the lack of a significant effect of PTX on N/L and P/L ratios in this population, chronically exposed to the effects of uremia.

In the TX group, a significant effect of PTX on PTH, calcium, phosphorus and vitamin D was also observed. Conversely, there was a reduction in the N/L and P/L ratios, at the expense of an increase in the lymphocyte population. A possible contributing factor to the effective lymphocyte response observed in this group, compared to HD group, is the impact of kidney transplantation on long-term systemic inflammatory status. In addition to the expected reduction in the accumulation of uremic toxins, kidney transplantation promotes a decrease in humoral inflammatory biomarkers and oxidative stress, such as interleukin-6, tumor necrosis factor alpha, and C-reactive protein^13^. However, the immunosuppressive therapies of transplant patients should be considered as confounding factors. All drugs currently used for maintenance therapy in kidney transplantation have direct or indirect effects on lymphocyte proliferation. Therefore, potential interference from these therapies cannot be excluded, as well as eventual changes to the dose or therapeutic regimen during the analyzed period^14^.

Therefore, based on our results, there was no change in N/L and P/L ratios among the SHPT population in the HD group after PTX. In contrast, in the TX group, there was a significant reduction in both ratios, possibly resulting from adjustments in maintenance immunosuppressive therapy.

The results of this study differ from those obtained by Yang et al.^15^, who observed a significant reduction in both N/L and P/L ratios in dialysis patients with SHPT undergoing PTX. However, it should be considered that the population evaluated in the aforementioned study had lower PTH concentrations than ours (1307 pg/mL), and were evaluated over a longer period (27 months). Other differences between the studied populations, which are difficult to measure, may also have influenced the results, such as hemodialysis quality, infections, and clinical management of CKD and comorbidities.

This study has several limitations. The great variation in the time elapsed between PTX and post-operative tests prevents us from excluding variations in the observed parameters by time elapsed after PTX. Furthermore, we cannot exclude the interference of different clinical events in the observed results, such as infections or decompensation of underlying diseases. It is also worth reiterating the aforementioned influence of immunosuppressive regimens used by the transplant population. Further studies, preferably prospective and controlled, are required to deepen our understanding on the subject. It is also necessary to expand the study of inflammatory biomarkers other than N/L and P/L ratios, including C-reactive protein (CRP) and inflammatory cytokines such as IL-6.
