# Supplementary material for: Analysis of neutrophil-to-lymphocyte and platelet-to-lymphocyte ratios as inflammatory biomarkers in chronic kidney disease: impact of parathyroidectomy
Source: J Bras Nefrol. 2024 Apr 8;46(3):e20230175. doi: 10.1590/2175-8239-JBN-2023-0175en (PMC11287880; doi:10.1590/2175-8239-JBN-2023-0175en)
Supplement: Translation

# Translation: Análise das relações neutrófilo/linfócito e plaqueta/linfócito como marcadores inflamatórios na doença renal crônica: impacto da paratireoidectomia

**DOI:** 10.1590/2175-8239-JBN-2023-0175pt

**Authors:** Andre Kakinoki Teng, Eduardo Jorge Duque, Shirley Ferraz Crispilho, Wagner Domingues, Vanda Jorgetti, Luciene M. dos Reis, Rosilene M. Elias, Rosa Maria Affonso Moysés

**Affiliations:** 1 Universidade de São Paulo, Faculdade de Medicina, São Paulo, SP, Brasil.; 2 Universidade Nove de Julho, São Paulo, SP, Brasil.

**Keywords:** Insuficiência Renal Crônica, Distúrbio Mineral e Ósseo na Doença Renal Crônica, Hiperparatireoidismo Secundário, Paratireoidectomia

## Abstract

**Introdução:**

O hiperparatireoidismo secundário (HPTS) é uma das causas de inflamação na DRC. Avaliamos o impacto da paratireoidectomia (PTX) nas relações neutrófilo/linfócito (N/L) e plaqueta/linfócito (P/L) em pacientes com HPTS.

**Métodos:**

Foram analisados 118 pacientes [hemodiálise (HD, n = 81) e transplantados (TX, n = 37)] submetidos à PTX entre 2015 e 2021.

**Resultados:**

Houve redução significativa de cálcio e PTH nos dois grupos, além de elevação de vitamina D. No grupo HD, a PTX não mudou as relações N/L e P/L. Já no grupo TX, houve redução nas relações N/L e P/L acompanhadas de elevação significativa do número de linfócitos totais.

**Conclusão:**

As relações N/L e P/L não são marcadores fidedignos de inflamação em pacientes com HPTS submetidos à PTX. A uremia, que induz um estado de inflamação crônica em pacientes dialíticos, e o uso de imunossupressão em pacientes transplantados renais são alguns dos fatores de confusão que impedem o uso dessa ferramenta na prática clínica.

## Introdução

A doença renal crônica está associada a um estado inflamatório sistêmico de origem multifatorial. Concentrações elevadas de paratormônio (PTH) e fator de crescimento de fibroblastos 23 (FGF-23), assim como as alterações de cálcio, fósforo e vitamina D observadas na doença mineral e óssea da DRC (DMO-DRC), possivelmente contribuem para essa condição^1,2^.

As relações neutrófilo/linfócito (N/L) e plaqueta/linfócito (P/L) são indicadores laboratoriais de baixo custo e alta acessibilidade, cujo valor como marcadores inflamatórios e prognósticos têm sido estudados em diferentes cenários, como em doenças oncológicas, cardiovasculares, infecciosas, entre outros^3,4^. A elevação dessas relações indica um aumento proporcional de células pró-inflamatórias (neutrófilos e plaquetas), em relação às reguladoras de resposta imune (linfócitos).

No contexto da DRC, estudos sugerem associação das relações N/L e P/L com inflamação, mortalidade cardiovascular e necessidade de terapia de substituição renal^5,6^. Tonyali et al.^7^ observaram associação da relação N/L com taxa de filtração glomerular (relação N/L 2,14 ± 0,73 em controles saudáveis *versus* 3,53 ± 2,3 no grupo com DRC, definida como TFG < 60 mL/min/1,73 m^2^, p = .000).

No entanto, há poucos estudos a respeito da utilidade desses marcadores no contexto específico da DMO-DRC. Em pacientes com hiperparatireoidismo secundário à DRC (HPTS), a paratireoidectomia (PTX) é uma opção de tratamento cirúrgico para os casos refratários. O objetivo deste estudo foi avaliar o papel da PTX nas relações N/L e P/L em pacientes com HPTS.

## Métodos

Este foi um estudo retrospectivo, em que foram analisados dados de prontuários de pacientes com HPTS submetidos à PTX em nosso serviço. Entre janeiro de 2015 e dezembro de 2021, 172 pacientes foram operados. Após exclusão dos casos sem dados laboratoriais disponíveis, ficaram 118 para análise. Foram registrados e analisados o hemograma completo, a contagem de plaquetas e as dosagens de cálcio total, cálcio iônico, fósforo, PTH e vitamina D séricos. Para os pacientes transplantados, foram coletados dados de creatinina sérica e taxa de filtração glomerular (TFG) pela fórmula CKD-EPI^8^. Para comparação, utilizamos como hemograma inicial aquele coletado imediatamente antes da data da cirurgia. O exame pós-PTX foi coletado em média 9 meses depois da cirurgia.

Os pacientes foram divididos em dois grupos, que foram analisados separadamente: pacientes transplantados renais (grupo TX) e pacientes em hemodiálise (grupo HD). Foram excluídos da análise 9 pacientes que receberam transplante renal durante o período estipulado entre os exames pré e pós-PTX.

Os dados contínuos foram apresentados como média ± desvio-padrão ou mediana e percentis (25; 75). A comparação entre grupos HD e TX foi feita por meio de teste t não pareado ou Mann-Whitney, de acordo com a distribuição dos dados paramétrica ou não, respectivamente. Para comparação antes e após a PTX, em ambos os grupos foram utilizados o teste t pareado ou Wilcoxon, para variáveis com distribuição normal ou não, respectivamente. A análise foi realizada usando o software GraphPad Prism 9.3.1^®^ software (GraphPad Software, Inc., CA, USA).

## Resultados

### Grupo Total

Foram analisados no total 118 pacientes, sendo 67 (56,7%) do sexo feminino. A idade média foi de 44,7 ± 13 anos. Conforme descrito na Tabela 1, houve queda do PTH, cálcio total e ionizado, acompanhada de elevação da concentração de 25-vitamina D. Não houve variação do fósforo sérico. Na análise de leucograma, houve aumento de leucócitos e linfócitos. A relação N/L não sofreu alteração significativa, mas observamos queda na relação P/L.

**Tabela 1:** Dados laboratoriais do grupo total

Grupo total	Pré-PTX	Pós-PTX	p
**Cálcio total (mg/dL)**	10,0 (9,3; 10,8)	8,8 (8,2; 9,6)	<0,0001
**Cálcio iônico (mg/dL)**	5,3 (4,9; 5,7)	4,6 (4,2; 5,1)	<0,0001
**Fósforo (mg/dL)**	4,6 (2,9; 6,1)	4,0 (3,1; 5,5)	0,1454
**PTH (pg/mL)**	1455 (493; 2081)	95 (51; 217)	<0,0001
**Vitamina D (ng/mL)**	27,0 (18,8; 32,4)	32,2 (26,4; 44,0)	<0,0001
**Leucócitos (céls./mm** ^3^ **)**	5900 (5000; 7900)	6400 (5175; 8125)	0,0358
**Linfócito (céls./mm** ^3^ **)**	1235 (1010; 1783)	1460 (1045; 1910)	0,0033
**Neutrófilos (céls./mm** ^3^ **)**	3920 (2945; 4963)	4185 (3150; 5465)	0,1028
**Plaquetas (10** ^3^ céls./mm^3^ **)**	190,0 (149,8; 243,3)	195,5 (172,8; 236)	0,0978
**Relação N/L**	2,88 (1,97; 3,97)	2,85 (2,12; 3,80)	0,3052
**Relação P/L**	143 (105; 194,7)	132,9 (102,4; 180,5)	0,0233

### Grupo HD

No grupo HD, foram analisados 81 pacientes, sendo 48 (59,2%) do sexo feminino. A idade média foi de 42,5 ± 13,6 anos. Conforme apresentado na Tabela 2, houve queda de cálcio total, cálcio iônico, fósforo e PTH; houve também elevação de 25-vitamina D. Na análise do leucograma, observou-se uma tendência a aumento nos leucócitos totais, mas não houve diferença significativa no total de linfócitos, neutrófilos e plaquetas. Não se observou alteração significativa das relações N/L e P/L (Figura 1).

**Tabela 2:** Dados laboratoriais dos grupos HD e TX

	Grupo HD			Grupo TX		
	Pré-PTX	Pós-PTX	Valor p	Pré-PTX	Pós-PTX	Valor p
**Creatinina (mg/dL)**	-	-	-	1,43 (1,11; 1,91)	1,46 (1,13; 1,70)	0,1801
**TFG (mL/min/1,73 m** ^2^ **)**	-	-	-	49 (36; 59)	50 (32; 61)	0,6101
**Cálcio total (mg/dL)**	9,6 (9,1; 10,4)	8,5 (7,6; 9,3)	<0,0001	10,5 (10,0; 9,4)	9,4 (9,0; 9,7)	<0,0001
**Cálcio iônico (mg/dL)**	5,0 (4,7; 5,4)	4,4 (4,1; 4,7)	<0,0001	5,7 (5,5; 6,2)	5,0 (4,9; 5,4)	<0,0001
**Fósforo (mg/dL)**	5,3 (4,5; 6,7)	4,8 (3,6; 5,9)	0,0002	2,5 (1,9; 2,9)	3,3 (2,7; 3,9)	<0,0001
**PTH (pg/mL)**	1695 (1417; 2509)	116 (43,5; 260)	<0,0001	294 (182; 536)	88 (57; 141)	<0,0001
**Vitamina D (ng/mL)**	29,0 (20,5; 35,05)	36,9 (28,4; 48,2)	<0,0001	22,6 (17,3; 28,7)	26,9 (25,1; 34,8)	0,0014
**Leucócitos (céls./mm** ^3^ **)**	5900 (5000; 8000)	6400 (4850; 8200)	0,069	5800 (4950; 7900)	6600 (5300; 8000)	0,2564
**Linfócito (céls./mm** ^3^ **)**	1370 (1060; 1950)	1500 (1150; 1855)	0,3137	1140 (795; 1645)	1280 (970; 1970)	<0,0001
**Neutrófilos (céls./mm** ^3^ **)**	3900 (2940; 4765)	4170 (2895; 5545)	0,1116	3940 (3015; 5605)	4210 (3365; 5285)	0,6146
**Plaquetas (10** ^3^ céls./mm^3^ **)**	198 (156; 248)	204 (175; 243,5)	0,202	174 (142; 201,5)	184 (158; 211,5)	0,2301
**Relação N/L**	2,7 (1,92; 3,61)	2,63 (2,12; 3,64)	0,4668	3,58 (2,09; 6,24)	2,97 (2,10; 4,36)	0,0123
**Relação P/L**	136,6 (101,8; 185,6)	131,7 (103,9; 177,4)	0,653	144,9 (110,7; 281,1)	138,6 (96,61; 193,2)	0,0011

**Figura 1.** Relações N/L e P/L pré e pós-PTX - Grupos TX e HD.

### Grupo TX

No grupo TX, foram analisados 37 pacientes, sendo 19 (51,3%) do sexo feminino. A idade média foi de 49,7 ± 9,8 anos. O intervalo médio entre o transplante renal e a PTX foi de 21 meses. Conforme descrito na Tabela 2, observou-se redução dos níveis séricos de PTH, cálcio total e cálcio iônico. Houve elevação dos níveis séricos de fósforo e 25-vitamina D. A taxa de filtração glomerular e a creatinina sérica não sofreram alteração significativa. Houve queda nas relações N/L e P/L (Figura 1). Tal resultado está associado a um aumento na contagem de linfócitos, sem alteração significativa do número de leucócitos, neutrófilos e plaquetas.

## Discussão

O HPTS é um dos fatores contribuintes para o estado inflamatório e disfunção imune celular observados em pacientes com DRC. O PTH é considerado uma toxina urêmica, com conhecidos efeitos, diretos e indiretos, sobre a inflamação, a função hematopoiética e a resposta imune adaptativa, tanto de linfócitos quanto de polimorfonucleares^9^. A PTX, como opção de tratamento para os casos refratários de HPTS, tem efeito sobre esse estado inflamatório, reduzindo diretamente os efeitos imunomoduladores do PTH.

No grupo HD, a PTX foi associada a redução significativa do PTH, cálcio e fósforo, além de aumento de vitamina D, conforme esperado, indicando sua eficácia como tratamento do HPTS. Porém, a análise dos resultados demonstrou que não houve alteração significativa das relações N/L e P/L nesse grupo. Tal resultado pode estar relacionado à persistência do estado inflamatório, de origem multifatorial, em pacientes com DRC. A uremia, por exemplo, interfere por meio de diversos mecanismos na resposta celular imune, tanto inata quanto adaptativa^10^.

O acúmulo de toxinas urêmicas está relacionado a um comprometimento da função e proliferação de linfócitos, que apresentam aumento de sua atividade apoptótica. Além disso, a exposição à uremia induz um processo de imunossenescência, similar ao que ocorre com o envelhecimento, com redução da atividade linfoproliferativa no timo^11^. A população de neutrófilos, por sua vez, aumenta à medida que a TFG cai. Observa-se, em pacientes em HD, aumento de espécies reativas de oxigênio, mieloperoxidase, e atividade de *priming*, demonstrando um estado de ativação inflamatória crônica^12^. O conjunto desses fenômenos possivelmente contribui para a ausência de efeito significativo da PTX sobre as relações N/L e P/L nessa população, exposta cronicamente aos efeitos da uremia.

No grupo TX, observou-se também efeito significativo da PTX sobre o PTH, cálcio, fósforo e vitamina D. Por sua vez, houve redução das relações N/L e P/L, às custas de um aumento na população de linfócitos. Um fator que possivelmente contribui para a resposta linfocitária efetiva observada nesse grupo, em comparação ao grupo HD, é o impacto do transplante renal no estado inflamatório sistêmico em longo prazo. Além da esperada redução do acúmulo de toxinas urêmicas, o transplante renal promove queda de marcadores inflamatórios humorais e de estresse oxidativo, como interleucina-6, fator de necrose tumoral alfa e proteína C reativa^13^. Contudo, é preciso considerar as terapias imunossupressoras dos pacientes transplantados como fatores de confusão. Todas as drogas atualmente usadas para a terapia de manutenção no transplante renal apresentam efeitos diretos ou indiretos sobre a proliferação linfocitária, de modo que não se pode afastar potenciais interferências dessas terapias e eventuais modificações de dose ou esquema terapêutico durante o período analisado^14^.

Portanto, diante dos nossos resultados, não houve alteração das relações N/L e P/L na população com HPTS no grupo HD após a PTX. Já no grupo TX, houve redução significativa de ambas as relações, possivelmente decorrentes de ajustes na terapia imunossupressora de manutenção.

Os resultados deste estudo diferem dos obtidos por Yang et al.^15^, que observaram redução significativa tanto da relação N/L quanto da P/L em pacientes com HPTS em diálise submetidos à PTX. No entanto, devemos considerar que a população avaliada no estudo citado apresentava concentrações de PTH menores do que a nossa (1307 pg/mL) e foram avaliados por um período mais prolongado (27 meses). Outras diferenças entre as populações estudadas, de difícil mensuração, também podem ter influenciado os resultados, tais como qualidade da hemodiálise, infecções e manejo clínico da DRC e comorbidades.

Este estudo apresenta diversas limitações. A grande variação no tempo decorrido entre a PTX e os exames pós-operatórios impede que excluamos variações nos parâmetros observados pelo tempo decorrido após a PTX. Além disso, não podemos afastar a interferência de intercorrências clínicas de diversas naturezas nos resultados observados, como infecções ou descompensações de doenças de base. Cabe também repetir a supracitada influência dos esquemas imunossupressores usados pela população transplantada. São necessários mais estudos, preferencialmente prospectivos e controlados, para aprofundar o entendimento sobre o tema. Também é necessário expandir o estudo de marcadores inflamatórios alternativos às relações N/L e P/L, tais como proteína C reativa (PCR) e citocinas inflamatórias, como IL-6.
